# The impact of epiphytic algae on the foliar traits of *Potamogeton perfoliatus*

**DOI:** 10.3389/fpls.2025.1561709

**Published:** 2025-04-24

**Authors:** Viktor R. Tóth

**Affiliations:** Aquatic Botany and Microbial Ecology Research Group, Hungarian Research Network Balaton Limnological Research Institute, Tihany, Hungary

**Keywords:** macrophyte, epiphyton, foliar morphology, foliar photophysiology, littoral zone, lake Balaton

## Abstract

This study investigated the effect of epiphyton on foliar traits of a submerged rooted macrophyte, *Potamogeton perfoliatus*, in a shallow freshwater lake, highlighting its influence on the ecological dynamics of littoral zones in aquatic ecosystems. It was shown that the limnological characteristics of the sampling sites (water chlorophyll-a, total suspended matter and coloured dissolved organic matter content) had no significant effect on the average values of epiphytic algal content found on pondweed leaves, while influencing the plasticity of these data. The responses of morphological and physiological traits of submerged macrophytes to accumulated epiphyton demonstrate the complexity of their relationship: epiphyton colonisation had no relevant effect on leaf morphology (except leaf length) and leaf pigment content (except Chl-a/Chl-b ratio), however, this study highlights the significant influence of epiphytic algal biomass on photophysiological traits of submerged macrophyte leaves, as 5 out of 6 photophysiological traits were affected. The results highlight the importance of considering epiphyte colonisation when seeking to understand the ecological functioning of littoral aquatic ecosystems. Furthermore, the complex interactions between epiphytes and submerged rooted macrophytes should be considered in integrated lake management and environmental protection policies. These interactions play an important, though ambiguous role in shaping habitat variability and overall ecosystem health in littoral zones.

## 1 Introduction

The dynamic relationship between epiphytes – a community of algae, microbes and fungi that colonise macrophyte surfaces – and submerged macrophytes is complex, and goes beyond the simple competition (Sand-Jensen and Søndergaard, 1981; Köhler et al., 2010; Wijewardene et al., 2022). It affects the structure and functioning of the littoral zone and can even influence the balance of the whole aquatic ecosystem, especially in shallow lakes. While both macrophytes and the autotrophic part of the epiphytic community play key roles by contributing to primary production, nutrient cycling, and habitat complexity (McAbendroth et al., 2005; Kovalenko et al., 2012; Tóth, 2024), their coexistence is often characterised by competition, notably for light (Sand-Jensen and Søndergaard, 1981; Vis et al., 2006; Sultana et al., 2010). Light is a primary resource required for photosynthesis, and in aquatic systems, it becomes increasingly limited with depth, turbidity, abundance of phytoplankton, epiphytes and macrophytes (Wetzel, 1975; Kirk, 1994; Tóth, 2013, 2024). Within macrophyte stands, light is attenuated by several factors (Kirk, 1994; Hill, 1996; Wijewardene et al., 2022). In particular, a significant proportion of the light attenuation reaching the leaf surface of submerged macrophytes is attributed to epiphytic algae (Tóth, 2013). Furthermore, the pigment systems of periphytic algae not only absorb light, thereby altering its quantity, but also do so selectively at specific wavelengths, thereby affecting the quality of the optical environment (Tóth, 2013, 2024; Klančnik et al., 2015; Tóth and Palmer, 2016). Individually, these phenomena could alter the morphology and physiology of aquatic plants, but their combined effects have the potential to induce significant phenotypic variability. Epiphytes often form dense layers on the leaves and stems of submerged plants, reducing the amount and quality of light reaching the macrophytes’ photosynthetic tissues, leading to physiological and morphological adjustments to compensate for these changes (Asaeda et al., 2004; Tóth, 2013, 2024).

For epiphytes, submerged macrophytes are primarily a substrate that maintains the community in an optimal environment, as aquatic plants provide essential habitat and structural complexity for various aquatic organisms (Kuczynska-Kippen, 2008; Thomaz et al., 2008). The presence of epiphytes on macrophyte surfaces further increases habitat complexity by providing additional food sources for micro- and macro-organisms (McAbendroth et al., 2005; Kovalenko et al., 2012; Tóth, 2013). However, when epiphyte growth becomes excessive, it can negatively affect macrophytes and reduce their capacity to perform their ecosystem services (Strand and Weisner, 1996; Köhler et al., 2010; Tóth, 2013) and thus could play a pivotal role in determining water clarity (Takamura et al., 2003; Thomaz, 2023). While macrophytes help to maintain clear water by reducing wave action, stabilising sediments, and sequestering nutrients (Søndergaard et al., 2010; Blindow et al., 2014), the weakened aquatic plants due to excessive epiphyte growth could disappear (Tóth, 2013; Wijewardene et al., 2022), and, over time, shift ecosystems towards a turbid, phytoplankton-dominated state (Scheffer et al., 1993; Scheffer and van Nes, 2007). Under conditions of nutrient enrichment, such as those seen in eutrophic systems, the increased growth of epiphytes can tip the balance against macrophytes, resulting in a shift towards degraded ecosystem states (Duffy et al., 2007; Özen et al., 2018). Conversely, stable macrophyte populations can help buffer ecosystems against the effects of eutrophication by sequestering nutrients and maintaining water clarity, demonstrating their role in ecosystem resilience (Duffy et al., 2007).

Competition between these two groups of autotrophs (epiphytes and submerged macrophytes) is particularly complex (Liboriussen and Jeppesen, 2006; Dos Santos et al., 2013; Tóth and Palmer, 2016), and while both require light and occupy nearly the same niche, the epiphytes have a significant advantage. The epiphytes can quickly respond to nutrient pulses and proliferate within days, while macrophytes, due to their complex structure and morphogenesis are slower to adjust to the changing conditions (Worm and Sommer, 2000; Yuan et al., 2023). There is an additional element to the temporal aspect of the interaction between macrophytes and their epiphytes. As epiphytic colonisation is a time-consuming process (the average colonisation time in Lake Balaton is around 8-9 days), the younger parts of the macrophytes are largely free of epiphytic cover (Tóth, 2013). Consequently, leaf morphogenesis in submerged macrophytes is initiated without the influence of epiphytes, and only gradually, over a period of time, does the epiphyte biomass begin to affect plant development. As epiphytes accumulate on the adaxial surface of leaves, their presence reduces the quantity and changes the quality of light to the photosynthetic tissues of the aquatic plant. To compensate for these changes, macrophytes may adjust their leaf morphology, physiology and resource allocation, potentially altering their vertical structure by promoting vertical expansion and branching in the apical parts (Tóth, 2024). In nutrient-rich conditions, where epiphyte loads are higher, macrophytes may show even more drastic changes, leading to the deterioration of basal parts of the plant.

Light perception involves different parts of the plant (Neff et al., 2000; Kami et al., 2010). However, the local information about the exact quantity and quality of light is perceived, expressed and only affects the growth and morphogenesis of the corresponding plant module at the specific leaf that sensed it (Quail, 2002; de Kroon et al., 2005; Tóth and Palmer, 2016). Consequently, submerged rooted macrophytes develop a morphological and physiological profile typical for their specific environment (Tóth, 2024).

The reduction in macrophyte abundance due to epiphyte overgrowth can have cascading effects on ecosystem services (Strand and Weisner, 1996; Köhler et al., 2010; Tóth, 2013). Submerged macrophytes play a critical role in stabilising sediments, enhancing water clarity, and providing habitat for a range of aquatic organisms. Their decline can lead to increased turbidity, altered nutrient cycling, and the loss of biodiversity, further pushing the system towards a turbid, phytoplankton-dominated state - a hallmark of advanced eutrophication.

Studying the interactions between epiphytes and macrophytes, therefore, provides insight into the broader processes that drive aquatic ecosystem change. As climate change exacerbates eutrophication by increasing nutrient loads and altering hydrological regimes, the need to understand these interactions becomes more urgent. By examining how epiphytes affect macrophyte foliar traits, physiological processes, and ecosystem functions, researchers can identify key factors that influence the resilience of aquatic plant communities. This knowledge is crucial for developing effective management strategies aimed at mitigating the impacts of eutrophication, preserving aquatic biodiversity, and maintaining the ecosystem services provided by submerged macrophytes.

Understanding the response of this underwater biotic framework (scaffold) to environmental conditions is crucial not only for comprehending the resilience and survival of macrophytes but also for understanding the ecology, interaction and phenology of organisms connected to these macrophytes. In order to assess the effect of epiphytic algae on the foliar traits of *Potamogeton perfoliatus L.* and their plasticity, the morphological and photophysiological characteristics of leaves at a certain depth were determined in different parts of Lake Balaton with different water quality parameters. The main questions were

a) which morphological leaf traits of *P. perfoliatus* are affected by epiphytic algae?
b) which photophysiological leaf traits of *P. perfoliatus* are affected by epiphytic algae?
c) how do studied limnological parameters affected leaf traits of *P. perfoliatus*?

## 2 Materials and methods

### 2.1 Lake Balaton

**Figure 1:**
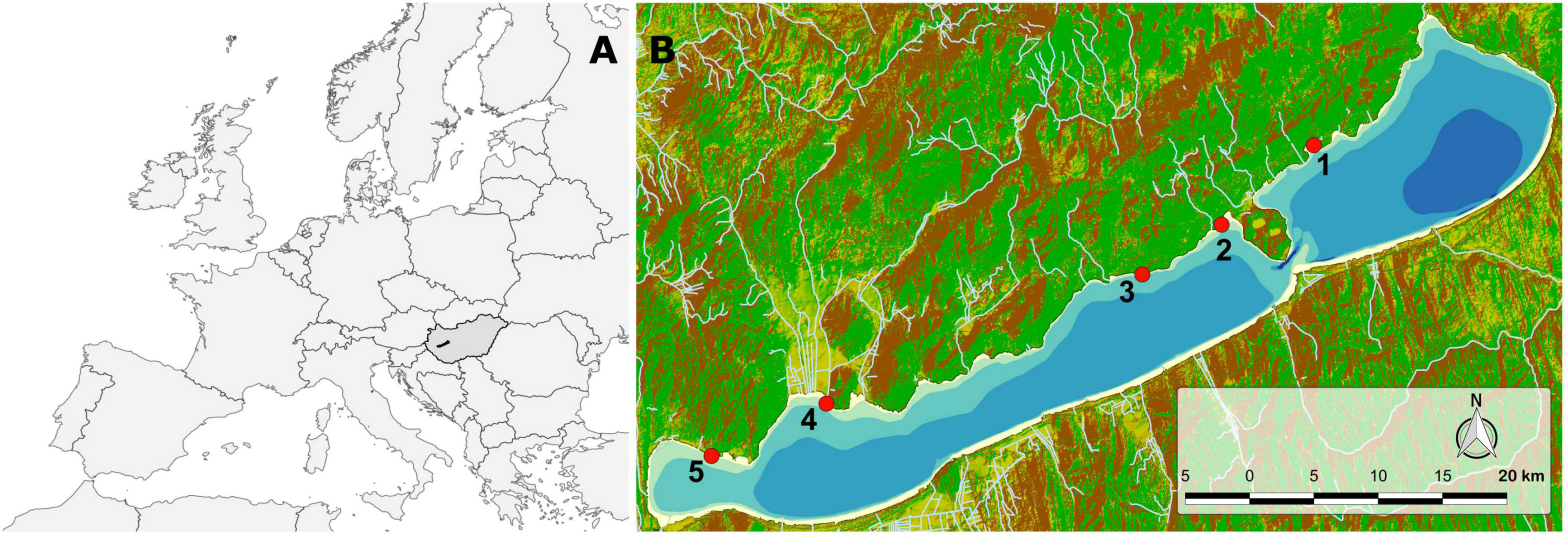
**(A)** Map of Europe showing Hungary (dark grey) and Lake Balaton (black polygon). **(B)** Topographic map of Lake Balaton and its surroundings, with sampling sites (red dots) along the northern shore. Green areas represent lowlands, yellow areas represent highlands, and brown areas represent mountains. Small tributaries of the lake are shown with white lines. The bathymetric areas of Lake Balaton are depicted with a 1-meter step. The darkest blue polygon in the easternmost part of the lake represents an area with waters deeper than 5 meters.

Lake Balaton (**Figure 1**) is a large (596 km²), shallow (mean depth 3.7 m) freshwater lake located in Central Europe (N 46.83°, E 17.71°). Due to its prevailing length (78 km), the lake can be divided into four basins, each of which has different limnological characteristics. The westernmost, Keszthely basin, historically has experienced eu-hypertrophic conditions at the end of summer, mainly due to considerable nutrient loading in the past. The easternmost, Siófok basin, is predominantly meso-oligotrophic. The prevailing north-westerly wind direction makes the southern shore exposed to waves, while the northern shore remains relatively calm. For convenience and feasibility of the sampling, observations and measurements were made in calm weather with only moderate wavelets on Lake Balaton.

### 2.2 Study areas

Experiments were conducted at five sites on the northern shore of Lake Balaton (**Figure 1**) at the phenological peak of the dominant species of Lake Balaton, the claspingleaf pondweed, *Potamogeton perfoliatus* L. (second half of July) in 2022. Site 1 is located in the easternmost meso-oligotrophic basin of Lake Balaton. Sites 2 and 3 are situated in the oligotrophic middle basin of the lake, while sites 4 and 5 are located in the eutrophic western and westernmost basins of the lake.

### 2.3 Water chemistry analyses

From each site, in the close vicinity of the studied pondweed stands depth-integrated water column samples were collected into one-litre bottles (3 from each site). The bottles were kept in a cooling box to maintain their original temperature and brought to the laboratory for further analysis within 20 minutes.

Total suspended matter (TSM) was measured gravimetrically after filtering the water through a previously dried and pre-weighed GF-5 fibreglass filter (nominal pore size = 0.4 µm), drying for 2 h at 105°C, then weighing again and subtracting the filter weight from the total weight. TSM concentration was calculated based on the predetermined volume of the filtered sample.

Coloured dissolved organic matter (CDOM) concentration was measured spectrophotometrically at 440 nm (Hitachi, U-2900) after filtering the water through a cellulose acetate filter (pore size 0.45 μm), and was expressed as Pt (platinum) units (mg Pt l^-1^) (Cuthbert and Del Giorgio, 1992).

Chlorophyll-*a* of the water column was determined from freshly collected samples. 1 litre of sampled water was filtered through Whatman GF-5 filter papers, and pigments were analysed following extraction in 60°C methanol and clarification by centrifugation (10 000 rpm for 10 min) (Iwamura et al., 1970).

### 2.4 Plant measurements

To ensure that the plants collected were of similar ecological status and age, a number of criteria were considered during sampling. At each site, 3-4 relatively large monospecific patches of clasping-leaf pondweed were selected along a 100-metre shoreline. To avoid self-shading, only patches with low to moderate densities (~5-6 plants per m²) were selected. Also, all selected stands were located in wave-exposed areas to reflect typical lake conditions. Sampled plants were located near the maximum depth of colonisation (1.8 m) and to maintain uniformity in plant age, all sampled pondweeds were 1.8 m long, i.e. their apical parts were at the water surface. Only individuals that were at least 1 metre from the edge of their macrophyte patch were sampled.

The sampling was caried out on foot. At each sampling site (as shown in **Figure 1B**), ~ 20 intact plants were chosen. One intact leaf was carefully collected from each of these *P. perfoliatus* plants at a depth of approximately 35 cm, roughly corresponding to the apical 25th leaf.

Leaves at a depth of 35 cm were chosen to ensure consistency of developmental stage and to minimise confounding variables related to plant physiology and environmental exposure. At this depth, the leaves sampled were among the youngest mature leaves, not yet entering senescence, but old enough for epiphytic algae to influence their morphological properties during morphogenesis. Previous experience in Lake Balaton has shown that epiphytic colonisation and foliar morphogenesis occurs in the same time: although epiphytic colonisation peaks after about 8 days (Tóth, 2013), leaves at 35 cm depth are 2 weeks old, i.e. biofilm accumulation was in its early stages, and these leaves were without epiphyton for part (probably half) of their development. Furthermore, the selected leaves were not deep enough to be affected by plant self-shading, ensuring that light availability remained a constant factor.

The stem was cut under and over the leaf, and the leaf then was carefully placed in a 20 ml glass vial not fully filled with purified water and sealed tightly. The vials were transported to the laboratory within an hour in the dark.

Upon arrival, in the laboratory, to remove any epiphyton adhering to the leaf surfaces the vials were shaken for 15 minutes. The leaves were then additionally gently washed and then transferred to 20 ml vials filled with filtered Lake Balaton water. The epiphyton removed from the leaves was filtered onto GFC filters (Whatman, USA). The filters were cut into small pieces to enhance the effectiveness of epiphytic algae pigment extraction. Epiphyton pigments present on these filters were extracted with an 80% acetone solution overnight at 4°C. To ensure that all pigments were removed from the filters, two additional extractions were performed in the morning. The supernatant’s absorbance was measured with a Shimadzu UV1600 spectrophotometer from Japan, and pigment concentrations were calculated using empirical equations (Wellburn, 1994).

The cleaned leaves were used first for photophysiological measurements and latter to determine their morphological properties. Chlorophyll fluorescence measurements were performed on these leaves within 1 hour of arrival to the laboratory. During this time the leaves were mostly kept in darkness. It started with the determination of the minimum fluorescence yield (F_0_) of the dark-adapted leaves. Subsequently, the maximum fluorescence yield (F_m_) was determined in leaves exposed to pulse-saturated light (λ=630 nm, I_s_=3000 μmol m^-2^ s^-1^) using a chlorophyll fluorimeter (PAM-2500, Heinz Walz GmbH, Germany). Following this initial assessment, each leaf was exposed to a sequence of 11 actinic illumination of known intensity ranging from 5 to 787 μmol m^-2^ s^-1^ (t=15 s, λ=630 nm). After each illumination step, both the apparent (F_s_) and light-adapted maximum (F_m′_) fluorescence values were measured with a new saturation pulse (λ=630 nm, I_s_=3000 μmol m^-2^ s^-1^). The apparent electron transport rate (ETR) was then calculated from the data obtained (**Table 1**) (Bilger and Schreiber, 1987; Schreiber et al., 1995; Schreiber, 1998).

**Table 1:** Fluorescence parameters calculated from PAM fluorometry.

parameter	name	equation	reference
*F_v_/F_m_*	maximum quantum efficiency of PSII	*(F_m_ − F_0_)/F_m_*	(Schreiber, 1998)
*qP*	photochemical quenching	*(F_m’_−F_s_)/(F_m’_−F_0′_)*	(Bilger and Schreiber, 1987)
*qN*	non-photochemical quenching	*1−(F_m’_−F_0’_)/(F_m_−F_0_)*	(Bilger and Schreiber, 1987)
*ETR*	electron transport rate	*(F_m’_−F_s_)/(F_m’_)·I·AF·0.5*	(Schreiber et al., 1995)

The equations contain the minimum (F_0_) and the maximum (F_m_) fluorescence yields, the apparent (F_s_) and maximum (F_m′_) values of fluorescence, the irradiance value (I), and empirical absorption factor (AF=0.84). For more information please see referenced literature.

Following the fluorescence measurement, each leaf was digitised using a scanner (CanoScan LiDE 60). Subsequently, from each digitised leaf, a 1 cm (diameter) disc was excised using a core borer. Leaf pigments were then extracted from the leaf discs, utilizing an 80% acetone solution overnight at 4°C. To ensure that all pigments were removed from the leaf disc, two additional extractions were performed in the morning. The absorption of the resulting supernatant was quantified using a spectrophotometer (UV1600, Shimadzu, Japan), and pigment concentrations were calculated by using empirical equations (Wellburn, 1994). The digital scans were processed to extract morphological traits of the leaves. This was done using ImageJ software (http://rsbweb.nih.gov/ij/), during which the length, width, area, perimeter, and circularity (ranging from 0 for an infinitely elongated polygon to 1 for a perfect circle) of the leaves were determined.

### 2.5 Mathematical and statistical analysis

The light response of the ETR was modelled with an exponential saturation curve (Eilers and Peeters, 1988). From the fitted model, the maximum electron transport capacity (*ETR_max_*), the theoretical saturation light intensity (*I_k_*) and the maximum quantum yield for the entire chain of electron transport (α) were retrieved.

The coefficient of variation was calculated for each specific trait at each sampling site to estimate trait plasticity. This parameter was considered equally important as the trait mean, as it provides insight into the extent of phenotypic flexibility, which is a crucial characteristic from an evolutionary perspective.

Statistical analyses were conducted using the R programming environment (R Development Core Team, 2012). Pearson product-moment correlation was applied to evaluate linear correlations between normally distributed variables, following the verification of normality through Shapiro-Wilk tests, with epiphytic algal chlorophyll concentration as an independent variable and morphological (length, width, area and circularity of leaves), photophysiological (*ETR_max_*, *I_k_*, α, F_v_/F_m_, qP and qN), and pigment (total chlorophyll concentration, chlorophyll a to b ratio, total chlorophyll to carotenoid ratio) parameters as dependent variables. When necessary, transformations were applied.

In preparing the Principal Component Analysis (PCA) plot, the data were first checked for normality and then normalised by centering each variable on its mean and scaling to unit variance. Following this normalisation, PCA was performed to extract the principal components that captured the majority of the variance in the data set. The first two components were then plotted in a biplot to visually represent the relationships between the morphological, photophysiological, pigmentation and limnological parameters. This procedure ensured that the influence of variables with different scales was minimised, thus accurately reflecting the inherent structure of the data.

Generalized additive models (GAM) were used to determine the effects of explanatory variables on epiphyton biomass. For one of the explored relationships the dependent variable was foliar epiphyton, while water chlorophyll-a, TSM, CDOM, and their interaction were used as continuous independent variables. For another explored relationship epiphyton (epi-chla), water chlorophyll-a content (w-chla), water total suspended matter (w-TSM), and water coloured dissolved organic matter (w-CDOM) were the independent, while leaf area, perimeter, circularity, length and width of leaves, the maximum quantum yield for whole-chain electron transport (α), maximum electron transport capacity (ETR_max_), the theoretical saturation light intensity (I_k_), maximum quantum efficiency of PSII (F_v_/F_m_), non-photochemical quenching (qN), photochemical quenching (qP), total chlorophyll (total chl), chlorophyll a to b ratio (chl_a_/chl_b_), total chlorophyll to carotenoid ratio (chl/car) were the dependent variables. The models used identity link functions with a Gaussian error distribution, as the data did not exhibit linear relationships. The Gaussian distribution was chosen to account for the continuous nature of the response variables, while allowing flexibility to capture non-linear patterns in the data (Hastie and Tibshirani, 1987; Wood, 2017). Prior to conducting the tests, normality of the data was assessed using the Shapiro-Wilk test. When necessary, transformations were applied. GAM was executed using the mgcv package (Wood, 2017).

## 3 Results

### 3.1 Effect of limnological parameters on epiphytic algal content

**Figure 2:**
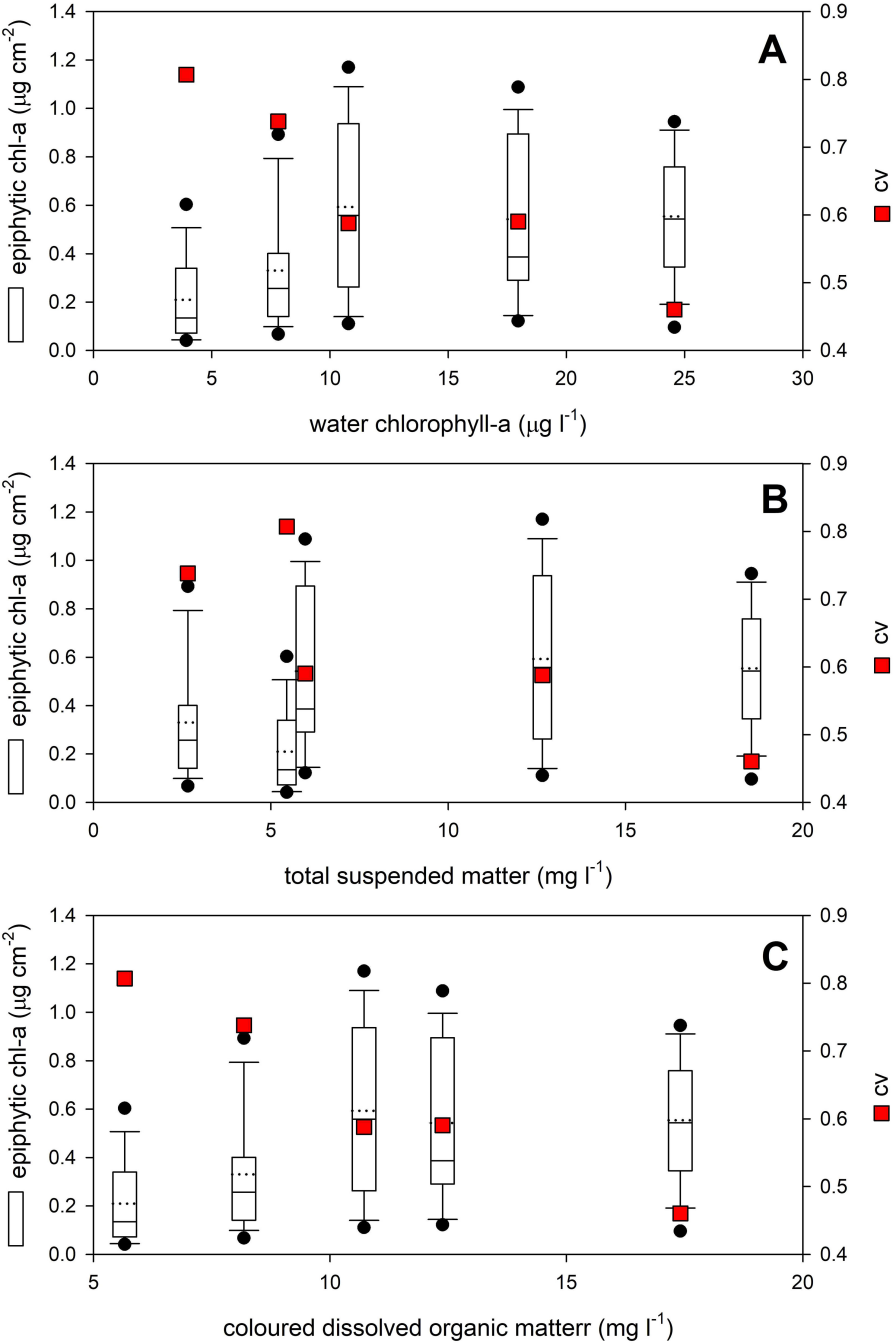
Effect of **(A)** phytoplankton (water chlorophyll-a), **(B)** total suspended matter and **(C)** coloured dissolved organic matter on the epiphytic content of *Potamogeton perfoliatus* leaves collected from 35 cm water depth. Data are shown with boxplots, while plasticity of data (cv - coefficient of variation) is shown as red squares.

The water quality of the studied areas, including the amount of phytoplankton, had no significant effect on the amount of epiphytic algal biomass found on *P. perfoliatus* leaves (**Table 2**, **Figure 2**). Each parameter had a non-linear, bitonic relationship with the epiphytic algal content, with the highest epiphytic chlorophyll-a content measured at 10.8 µg l^-1^ water chlorophyll-a content, at 12.6 mg l^-1^ total suspended matter and 10.7 mg l^-1^ coloured dissolved organic matter (**Figure 2**, white circles).

**Table 2:** Summary results of the generalized additive models (GAM) for amount of epiphyton (epiphytic algal chlorophyll-a) in the studied sites described by chlorophyl-a content (chl-a), total suspended matter (TSM), and coloured dissolved organic matter (CDOM) of the water.

	Estimate	Std. Error	t value	Pr(>|t|)
Intercept	4.6313	0.2765	16.75	<2*10^-16^
	edf	Ref.df	F	p-value
s(chl-a)	1.056	1.062	0.012	0.980
s(TSM)	1.056	1.062	0.409	0.598
s(CDOM)	1.783	1.865	2.362	0.111
R-sq.(adj)	0.18			
Deviance explained	21.1%			
GCV	8.4228			

The GCV score is the minimised generalised cross-validation (GCV) score of the GAM fitted.

Contrary to this, plasticity of the epiphytic algal content showed monotonic relationship with the studied limnological parameters (**Figure 2**, red squares). The highest plasticity of the epiphytic algal content was recorded at the lowest values of the studied limnological parameters and the plasticity was gradually decreasing with increase of every studied limnological parameter resulting in Pearson product-moment correlation of *r*=-0.94 (*p*=0.019), *r*=-0.84 (no significance), and *r*=-0.97 (*p*=0.005) for water chlorophyll-a content, total suspended matter and coloured dissolved organic matter, respectively (**Figure 2**, red squares).

### 3.2 Effect of epiphytic algal biomass on foliar traits

The coefficient of variation (CV) data obtained from the samples were very different, providing some background information on the relationship between the epiphyton and the morphological and photophysiological parameters studied. The chlorophyll-a content of the epiphyton had the highest CV (67.2 ± 14.4%), indicating considerable plasticity in the epiphytic algal biomass within the study sites, while the morphological, photophysiological and pigment parameters varied much less. The morphological parameters showed the lowest CV (17.6 ± 5.8%) plasticity, indicating a more stable response, a consistent structural development across sampling sites, regardless of the differences in epiphytic biomass. The physiological parameters, with a CV of 18.8 ± 8.4%, showed a similar plasticity as the morphological characteristics. Foliar pigment parameters had a CV of 25.3 ± 7.5% (mainly due to the high plasticity of the chlorophyll:carotenoid ratio (~32%). This suggests that pigment adjustments, possibly related to changes in light conditions due to epiphyton shading, were somewhat variable, but less so than the periphyton biomass itself.

**Figure 3:**
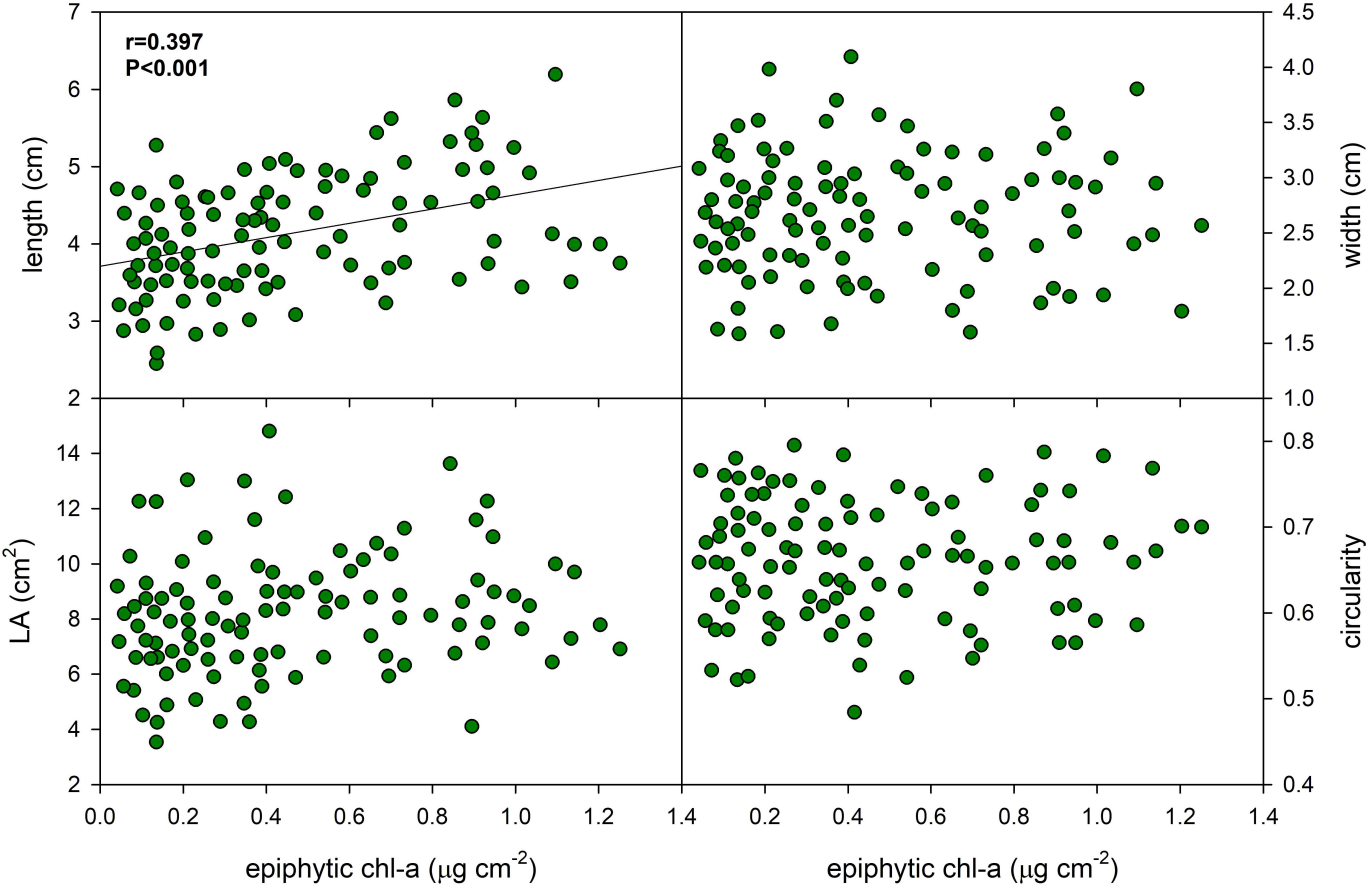
Effect of epiphyton content on the foliar morphology of *Potamogeton perfoliatus* collected from 35 cm deep water. The numbers within the graph show the Pearson product moment correlation and its significance. Correlations and significance levels are given for significant relationships (p<0.5).

Epiphyton biomass accumulated on the of *P. perfoliatus* leaves had little effect on leaf morphology (**Figure 3**, **Table 3**). The data indicate a moderate positive linear relationship between epiphyton biomass and leaf length in *P. perfoliatus* (Pearson product moment correlation coefficient *r*=0.397, *p*<0.001), resulting in 23% increase of leaf length, suggesting that the conditions of epiphyton biomass accumulation are also favourable for leaf longitudinal growth, while other morphological parameters (leaf width, leaf area, leaf circularity and leaf perimeter) were not affected in the same way (**Figure 3**).

**Table 3:** Summary results of the generalized additive models (GAM) for the foliar morphological and photophysiological properties of *Potamogeton perfoliatus* at 35 cm depth affected by the amount of epiphyton (epi-chla), water chlorophyll-a content (w-chla), water total suspended matter (w-TSM), and water coloured dissolved organic matter (w-CDOM) of the studied sites.

	intercept	epi-chla	w-chla	w-TSM	w-CDOM
leaf area	2.92^**^	2.02^*^	-1.67	-0.14	1.14
perimeter	6.56^***^	1.34	-0.38	-0.81	0.29
circularity	8.27^***^	0.27	-0.22	0.28	0.02
length	4.64^***^	4.20^***^	-0.93	-0.52	0.79
width	4.08^***^	-0.01	-0.67	-0.84	0.74
α	5.36^***^	4.86^***^	0.05	-0.27	-0.03
ETR_max_	2.62^*^	-3.72^***^	-2.87^**^	-3.03^**^	2.92^**^
I_k_	0.85	-4.17^***^	-3.22^**^	-3.34^**^	3.34^**^
F_v_/F_m_	10.75^***^	2.93^**^	1.03	0.29	-0.87
qN	11.73^***^	-1.18	0.65	0.22	-0.58
qP	1.83	-4.52^***^	-3.09^**^	-2.63^**^	3.03^**^
total chl	6.35^***^	0.73	1.51	1.78	-1.64
chl_a_/chl_b_	3.29^**^	-3.32^**^	-1.00	-0.47	0.93
chl/car	3.13^**^	-1.87	-0.45	0.12	0.47

Data showed the t value of the test and its significance. The shown parameters are the leaf area, perimeter, circularity, length and width of leaves, the maximum quantum yield for whole-chain electron transport (α), maximum electron transport capacity (ETR_max_), the theoretical saturation light intensity (I_k_), maximum quantum efficiency of PSII (F_v_/F_m_), non-photochemical quenching (qN), photochemical quenching (qP), total chlorophyll (total chl), chlorophyll a to b ratio (chl_a_/chl_b_), total chlorophyll to carotenoid ratio (chl/car). The significance of the GAM tests: * - *p*<0.05, ** - *p*<0.01, *** - *p*<0.001.

**Figure 4:**
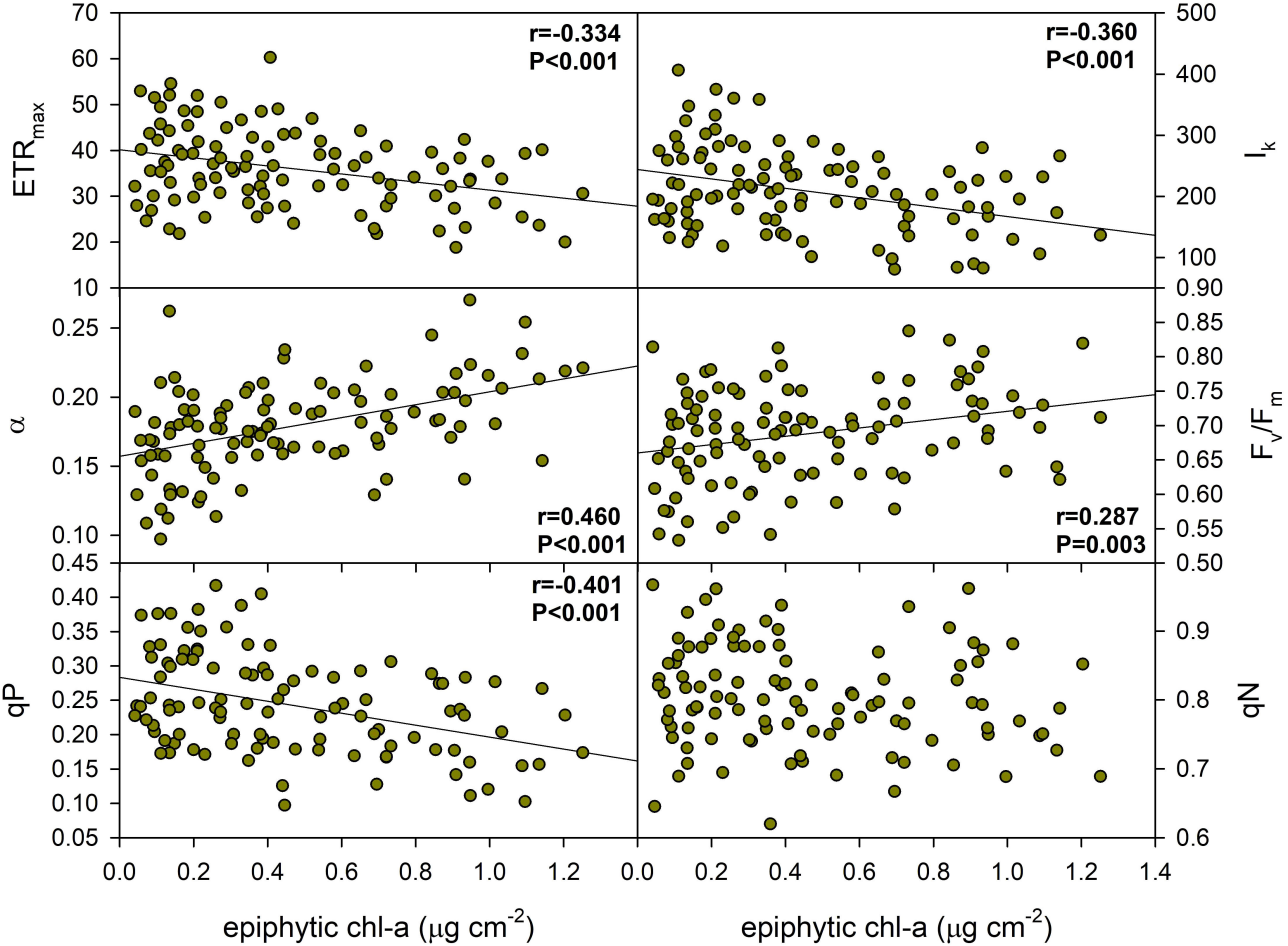
Effect of epiphyton on the photochemical properties of *Potamogeton perfoliatus* collected from 35 cm deep water. The numbers within the graph show the Pearson product moment correlation and its significance. Correlations and significance levels are given for significant relationships (p<0.5).

The results suggest that epiphyton biomass had a significant, but moderate effect on several aspects of the photophysiology of *P. perfoliatus* leaves, affecting the initial slope of the photosynthesis-light curve (α), the minimum saturation irradiance (I_k_) and the maximum relative electron transport rate (ETR_max_), the maximum efficiency of PSII (F_v_/F_m_) and the photochemical quenching (qP) of PSII (**Figure 4**, **Table 3**). ETR_max_, I_k_ and qP were negatively correlated, indicating a decrease in maximum electron transport rate (37.5%), minimum saturation irradiance (62.7%) and photochemical quenching (59.4%) with increasing epiphyton biomass. Meanwhile, α and F_v_/F_m_ showed a positive increase of 24.5% and 9.7%, respectively, with an increase in leaf epiphyton biomass (**Figure 4**).

**Figure 5:**
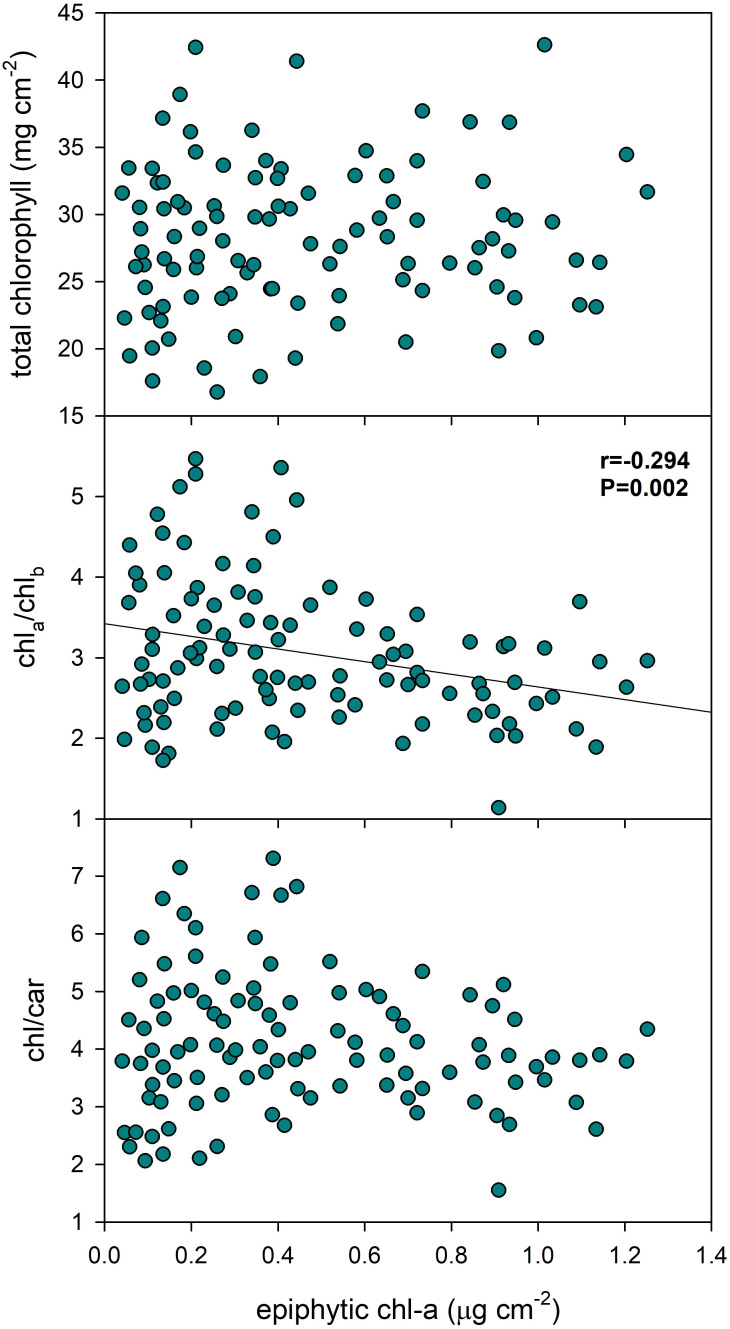
Effect of epiphyton on the pigment content of *Potamogeton perfoliatus* leaves collected from 35 cm deep water. The numbers within the graph show the Pearson product moment correlation and its significance. Correlations and significance levels are given for significant relationships (p<0.5).

Epiphyton biomass exhibited a low, though statistically significant negative correlation (Pearson product moment correlation: *r*=-0.294, *p*=0.002) with the Chl-a/Chl-b ratio in *P. perfoliatus* leaves, indicating a decrease in the ratio of chlorophyll-a to chlorophyll-b with higher epiphyton biomass (**Figure 5**, **Table 3**). Removing the outliers (chl a/b > 4.5 and < 1.5) did not substantially reduce the Pearson product-moment correlation, which remained significant (r = -0.246, p = 0.015). However, epiphyton biomass did not show a statistically significant effect on total chlorophyll content or the chlorophyll:carotenoid ratio in the data set examined.

**Figure 6:**
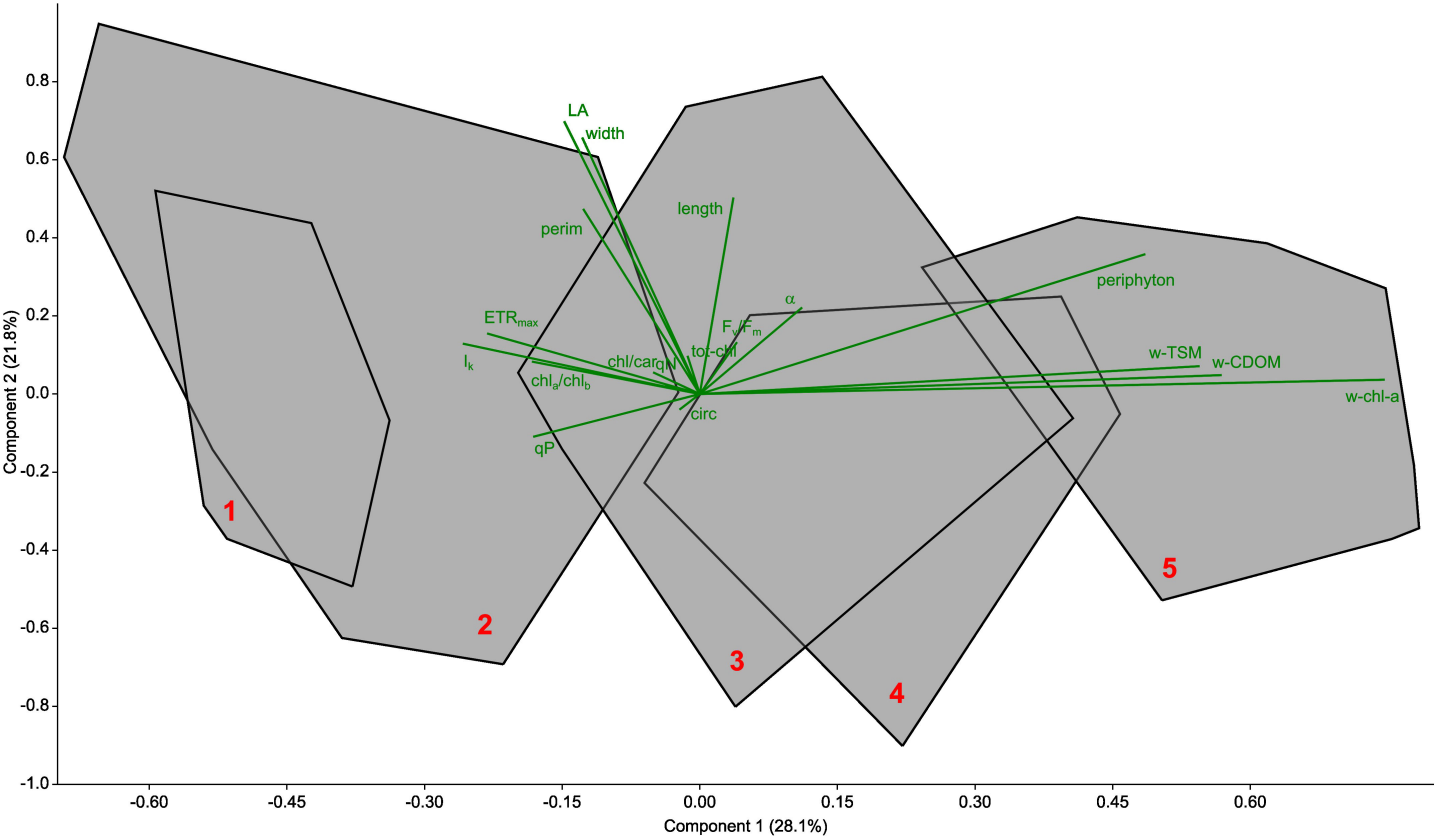
Principal component analysis (PCA) biplot of all parameters determined from the Lake Balaton sampling. Data from each site are grouped into a convex hull (data points not shown) and marked with a red number. The biplot is shown in green. The biplot shows the studied limnological (wTSM – total suspended matter of the water, wCDOM – coloured dissolved organic matter of the water, wchl-a – chlorophyll-a content of the water), morphological (length, width, LA – area, perim – perimeter and circ - circularity of the leaves), photophysiological (ETR_max_ - the maximum electron transport capacity, I_k_ - the theoretical saturation light intensity, the α - maximum quantum yield for the entire chain of electron transport, F_v_/F_m_, - maximum quantum efficiency of PSII, qP - photochemical quenching, qN - non-photochemical quenching), pigment (totchl - total chlorophyll concentration, chl_a_/chl_b_ - chlorophyll a to b ratio, chl/car - total chlorophyll to carotenoid ratio) and periphytic (periphyton – epiphytic chlorophyll-a content) parameters.

Principal component analysis (**Figure 6**) revealed that the differentiation between sampling sites was primarily driven by differences in periphyton biomass, total suspended matter (TSM) and coloured dissolved organic matter (CDOM). These factors had the greatest influence on site separation. In addition, physiological parameters such as maximum electron transport rate (ETR_max_), light saturation point (I_k_) and photochemical quenching (qP), as well as pigment properties, in particular the chlorophyll a:b (Chl-a/Chl-b) ratio and the chlorophyll:carotenoid ratio, contributed to the differentiation. These physiological and pigmentary traits, although secondary in influence to periphyton and limnological parameters, also played a role in the observed separation of the sites, indicating their relevance in characterising foliar traits and environmental conditions across sites (**Figure 6**).

**Figure 7:**
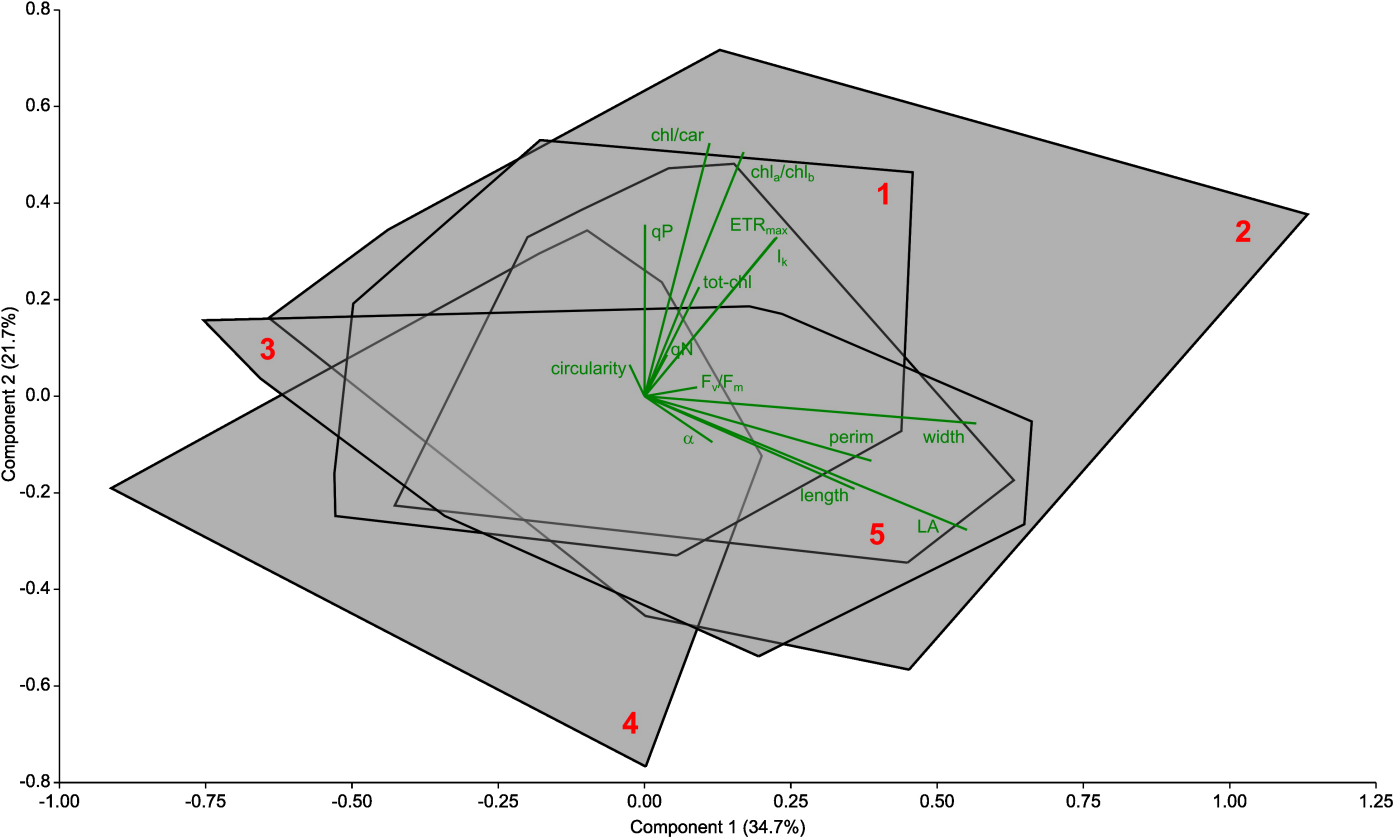
Principal component analysis (PCA) biplot of the determined foliar morphological, photochemical and pigment parameters collected from *Potamogeton perfoliatus* plants at 35 cm deep waters of Lake Balaton. Data from each site are grouped into a convex hull (data points not shown) and marked with red number. Trait biplot show in green colour. The biplot shows the studied morphological (length, width, LA – area, perim – perimeter and circ - circularity of the leaves), photophysiological (ETR_max_ - the maximum electron transport capacity, I_k_ - the theoretical saturation light intensity, the α - maximum quantum yield for the entire chain of electron transport, F_v_/F_m_, - maximum quantum efficiency of PSII, qP - photochemical quenching, qN - non-photochemical quenching) and pigment (totchl - total chlorophyll concentration, chl_a_/chl_b_ - chlorophyll a to b ratio, chl/car - total chlorophyll to carotenoid ratio) parameters. No significant differences detected (P>0.05).

The PCA performed only on the morphological, photophysiological and pigment content parameters of the studied *P. perfoliatus* leaves did not significantly distinguish the studied sites in Lake Balaton (**Figure 7**), suggesting that the lack of territorial response shows a core species-level response to epiphytic colonisation.

The relations shown on **Table 3** suggest that epiphyton accumulated on the leaf surface of *P. perfoliatus* is associated with certain aspects of leaf morphology (weakly positive with leaf length and area). Certain photophysiological parameters (positive with alpha, F_v_/F_m_ and negative with ETR_max_, I_k_ and qP) and certain parameters of leaf pigment content (negative with Chl-a/Chl-b ratio) were also associated by the GAM with epiphyton biomass (**Table 3**).

## 4 Discussion

From a meteorological and limnological point of view, the study year (2022) at Lake Balaton was not considered exceptional. No local algal blooms were observed at the study sites in the weeks prior to the survey, and no major storms occurred in July. While environmental background of epiphyton accumulation is complex and is often influenced by factors such as nutrient levels, light availability, and the composition of dissolved organic matter (DOM) (Aizaki and Sakamoto, 1988; Frost et al., 2007), this work found no significant correlation between the major limnological parameters, including the amount of phytoplankton in the water, and epiphytic algal biomass in the studied sites of Lake Balaton. This lack of relationship further confirmed the non-linear association between water quality and periphyton accumulation as previously reported (Hansson, 1992; Lowe, 1996; Liboriussen and Jeppesen, 2003). At low values of the limnological parameters studied, the amount of epiphytic algae remained low and increased only up to a certain threshold (water chl-a at 18 µg l^-^¹, TSM at 15 mg l^-^¹ and CDOM at 14 mg l^-^¹), beyond which other factors, such as light attenuation, influenced the relationship and changed its nature.

For the epiphyte, the submerged macrophyte is mostly a substrate, an available and useful structure to be exploited. The accumulation of epiphyton on the surface of broad-leaved submerged macrophyte leaves can have both direct (light attenuation, nutrient competition) and indirect (community restructuring, light quality filtering, etc.) effects (Brandt and Koch, 2003; Tóth, 2013; Tóth and Palmer, 2016; Wijewardene et al., 2022). Although only one component of the epiphyton, the epiphytic algae, was quantified in this study, the relatively young age of the leaves was chosen on the assumption that only algae had colonised the leaves. In reality, no prior information on this aspect was available at the time, as studies on the dynamics of epiphyton accumulation in Lake Balaton are still lacking. Nevertheless, this study showed direct evidence that epiphytic algal content is affecting foliar traits of pondweeds in Lake Balaton. Although the observed relationships were weak, some correlations were as high as 0.45 and explained nearly 25% of variance, a clear association between epiphytic algal content and the studied leaf traits of pondweed was evident.

It was shown that, apart from contributing to an increase in leaf length, epiphytic biofilms had no significant effect on the leaf morphology of the studied pondweeds. This finding is partially consistent with previous studies indicating that low light conditions can induce compensatory changes in leaf morphology (Goldsborough and Kemp, 1988; Tóth et al., 2011). The robust and time-intensive nature of leaf photomorphogenesis may underlie this phenomenon, as the younger, developing leaves - located at the apical part of the pondweed - were able to respond to changing environmental conditions. Over time, epiphyte colonisation probably altered the initial conditions, leading to secondary morphogenetic transformations. These secondary changes may have counteracted the primary responses, ultimately resulting in the observed weak correlations between epiphyton density and leaf morphological traits.

Pigment photomorphogenesis is a rapid but long-lasting (from days to weeks) response to various environmental signals (Barko and Filbin, 1983; Horppila et al., 2022; Tóth, 2024): chlorophyll molecules are continuously synthesised and degraded within chloroplasts, while their lifetime depends on factors such as light exposure, temperature and plant age. This study found no correlation between the amount of epiphytic algae and the total chlorophyll content or chlorophyll to carotenoid ratio of the leaves. However, a weak negative correlation was observed between the epiphyton found on the leaves and the Chl-a/Chl-b ratio of these leaves: the leaves with no, or minimal epiphyton had 25% more chlorophyll-a molecules, compared to leaves with high epiphytic algal biofilm. This response mechanism of leaf pigments to epiphyton could be connected to adaptive biochemical adjustments in pigment composition as a response to spectral changes of light reaching the photosystem of leaves (Tóth and Palmer, 2016; Tóth, 2024), since in low light environments, plants often increase the size of their light-harvesting antennae to capture more light, thereby adjusting the chlorophyll a:b ratio (Dale and Causton, 1992; Murchie and Horton, 1997; Beneragama and Goto, 2010).

Changes in foliar photophysiological traits require several complex metabolic pathways, but are easily reversed and thus may be part of the short-term, fast-response acclimation tactics of submerged macrophytes. The photophysiological responses to epiphytic algae were more pronounced, as 83% of the traits studied showed a relationship to epiphytic algae, whereas only 40% of morphological and 33% of pigment traits responded to the same conditions, although it cannot be excluded that these effects result from the combined influence of the entire epiphyton community. The negative correlations observed for ETR_max_, I_k_ and qP and the positive correlations observed for α and F_v_/F_m_ suggest that higher epiphyton biomass may affect the photosynthetic efficiency and capacity of leaves, possibly due to light attenuation. Not only the magnitude of photophysiological responses (83%) to epiphyton was substantial, but the photophysiological traits studied also showed high plasticity of responses (27% on average) to epiphytic algal content compared to the plasticity of morphological (11% on average) and pigment (13% on average) traits, demonstrating the immediate involvement of the Photosystem II complex in the process. Therefore, the morphological and pigment responses could be estimated to be used as an adaptive response of plants, whereas photophysiological responses are faster, more plastic and cost-effective way to acclimate to changing environmental conditions. The whole framework for understanding the differential responses of macrophytes to epiphytic algal abundance is based on the interplay between immediate plasticity and strategic adaptive responses in shaping macrophyte fitness and ecological resilience.

Regarding the mechanism of action, in the case of submerged rooted macrophytes, which take up nutrients mainly from the sediment and where the effect of epiphyton on the nutrient content of the foliage could be considered negligible, the primary mechanism of action of epiphyton in the case of pondweeds is through reduced light penetration, potentially limiting the photosynthetically available leaf area, limiting gas exchange and affecting photosynthesis in general (Tóth, 2013; Klančnik et al., 2015). Epiphyton especially its algal component can impair leaf physiology, thus changing the survival strategies of macrophytes, and could be the first factor resulting in the disappearance of macrophyte during eutrophication (Scheffer and van Nes, 2007; Tóth and Palmer, 2016). Epiphyton may also affect macrophytes by altering the spectral composition of light reaching the macrophyte leaf surface. Biofilm structure and pigmentation selectively filter incident light, potentially attenuating more ultraviolet (UV) radiation while allowing the transmission of photosynthetically active radiation (PAR) (Tóth, 2013). This selective filtering could act as a protective mechanism, reducing photoinhibition and photodamage under high light conditions, which may be relevant in shallow and near-surface waters. In addition to modulating light availability, epiphytes may also influence the local availability of inorganic carbon to macrophytes. The presence of epiphytic biofilms could modify the local carbon environment by affecting diffusion dynamics and altering the carbonate balance at the leaf surface. This modification could occur through metabolic activities such as respiration and photosynthesis within the biofilm matrix, which could create microgradients of dissolved inorganic carbon (DIC) (Sand-Jensen et al., 1985; Madsen et al., 1993). These complex interactions should also be considered when assessing the ecological role of epiphyton in macrophyte physiology and growth.

This study investigated the effect of epiphyton on foliar characteristics of submerged rooted macrophytes. Despite the influence of other seemingly more important factors such as herbivory, light availability, water clarity, species composition and macrophyte biomass, the weak but significant effects of epiphyton on plant physiology and morphology highlight its importance in the functioning and biodiversity of the littoral zone. Although epiphyton encompasses more than photosynthetic algae, this study specifically assessed the effects of epiphytic algae and may have underestimated the wider importance of epiphyton. It was shown that aquatic plants can employ a wide range of adaptive responses to ensure their survival, depending on the prevailing conditions. The response to epiphyton colonisation varied from significant to negligible, highlighting the need to consider this interaction to fully understand the littoral zone. These results highlight the importance of integrated lake management and environmental protection strategies that take into account the complex interactions between epiphytes and submerged rooted macrophytes. Recognising the dynamic nature of these interactions and their effects on habitat structure, biodiversity and ecosystem functioning could guide management efforts to mitigate anthropogenic impacts and enhance the resilience and sustainability of aquatic environments.

## Data Availability

The raw data supporting the conclusions of this article will be made available by the authors, without undue reservation.
